# Leveraging pre-trained embeddings in an ensemble machine learning approach for Arabic sentiment analysis

**DOI:** 10.3389/frai.2025.1653728

**Published:** 2025-09-11

**Authors:** Areej Jaber, Israa Bahati, Paloma Martínez

**Affiliations:** ^1^Computer Science Department, Palestine Technical University - Kadoorie, Tulkarm, Palestine; ^2^Computer Science and Engineering Department, Universidad Carlos III de Madrid, Leganes, Spain

**Keywords:** ensemble learning, sentiment analysis, machine learning, Arabic language, SMOTE

## Abstract

**Introduction:**

Arabic sentiment analysis presents unique challenges due to the linguistic complexity of the language, including its wide range of dialects, orthographic ambiguity, and limited language resources. Addressing these issues is essential to develop robust sentiment classification systems.

**Methods:**

This study investigates the application of ensemble machine learning methods for Arabic sentiment analysis. Several homogeneous ensemble techniques are implemented and evaluated on two datasets: the balanced ArTwitter dataset and the highly imbalanced Syria_Tweets dataset. To mitigate class imbalance, the Synthetic Minority Over-sampling Technique (SMOTE) is employed. The models incorporate pre-trained word embeddings and unigram features.

**Results:**

Experimental results indicate that individual classifiers using pre-trained embeddings achieve strong performance; however, ensemble models consistently yield superior outcomes. On the ArTwitter dataset, the ensemble of Naive Bayes, Support Vector Machine, and Decision Tree classifiers achieved an accuracy of 90.22% and an F1-score of 92.0%. On the Syria_Tweets dataset, an ensemble combining Stochastic Gradient Descent, k-Nearest Neighbors, and Random Forest attained 83.82% accuracy and an 83.86% F1-score.

**Discussion:**

The findings highlight the effectiveness of ensemble learning in enhancing the robustness and generalizability of Arabic sentiment analysis systems. Incorporating pre-trained embeddings further strengthens performance, demonstrating that ensemble-based approaches can overcome challenges posed by linguistic complexity and dataset imbalance in Arabic natural language processing tasks.

## 1 Introduction

With recent advancements in Natural Language Processing (NLP), several text analysis tasks have been successfully automated, including disinformative tweets detection (Jaber and Mart́ınez, 2023), word sense disambiguation (Jaber and Mart́ınez, 2022), and propaganda detection (Duridi et al., 2025). Sentiment analysis, a subtask of text classification, aims to classify a piece of text into binary classes (positive or negative) or multi-class categories (positive, negative, neutral). It has found widespread application across various domains, including politics (Grover et al., 2025), business (Tiwari and Arora, 2025), and social media (Alotaibi et al., 2025).

The performance of sentiment analysis systems largely depends on two core phases: feature engineering and the choice of classification algorithms. Feature engineering refers to transforming raw textual data into numerical representations that capture the semantic and syntactic properties of the text. Traditional approaches such as Term Frequency- Inverse Document Frequency (TF-IDF) and n-gram models have been effective in handling short texts (Nafis and Awang, 2021). More recent approaches based on word embeddings, including Word2Vec (Church, 2017), GloVe (Pennington et al., 2014), FastText (Joulin et al., 2016), and Large Language modeling (Mansour et al., 2025) provide rich semantic context and reduce the sparsity problem inherent in high-dimensional representations.

Among the classification strategies, ensemble learning has shown great promise in improving NLP task performance. The key idea of ensemble methods is to combine the predictions of multiple base classifiers to offset the weaknesses of individual models while leveraging their strengths. Ensemble learning based on machine learning algorithms has demonstrated its effectiveness across various NLP applications (Rane et al., 2024).

Arabic is one of the six official languages of the United Nations and is the native language of over 300 million people across 22 countries. However, Arabic sentiment analysis poses numerous challenges due to the linguistic complexity of the language. These challenges include morphological richness, the presence of multiple dialects, and the frequent use of figurative language such as ambiguity, sarcasm, and irony (Rahma et al., 2023), which makes sentiment classification more difficult (Alwakid et al., 2017).

The contribution of this work is an model based on a majority voting homogeneous ensemble machine learning approach. Exploring different vector-based feature representations and machine learning algorithms, including TF-IDF with ngrams and pretrained word embeddings. To address the issue of class imbalance during training, the Synthetic Minority Oversampling Technique (SMOTE) is employed Syria_tweet dataset. Optimize the hyperparameters of the proposed model to achieve the highest possible classification performance. The results are compared with the most relevant previous work, which demonstrates its superior performance.

The remainder of this article is organized as follows: Section 2 reviews prior studies on dialectal Arabic sentiment classification. Section 3 presents the proposed research methodology. Section 4 discusses the experimental results and evaluations. Finally, Section 5 concludes the study and outlines directions for future research.

## 2 Related work

Sentiment analysis has become quite popular in many languages, including Arabic, since social media, product evaluations and opinions, and user-generated content are becoming more and more important. Several comprehensive surveys have traced the evolution of Arabic sentiment analysis and mapped out the key resources in the field. (Ghallab et al. 2020) reviewed work published between 2015 and 2019, grouping existing approaches into three main categories: lexicon-based, machine learning-based, and hybrid methods that combine the two. Their review also provided an overview of more than twenty available datasets, ranging from domain-specific corpora to large Twitter-based collections such as ASTD and ArSenTD-Lev, which remain popular because of Twitter's rich mix of short, informal, and often dialectal content.

A more focused perspective was offered by (Obiedat et al. 2021), who surveyed research on **Arabic aspect-based sentiment analysis (ABSA). Their study covered early rule-based and lexicon methods, as well as more recent deep learning architectures that integrate pre-trained embeddings and attention mechanisms. They also listed key ABSA resources, including the SemEval Arabic corpora and HARD, and discussed persistent challenges such as handling the diversity of Arabic dialects, the scarcity of large annotated datasets, and the difficulty of building models that generalize well across domains.

Sentiment analysis approaches can be categorized into three categories: lexicon-based approaches, machine learning approaches, and hybrid approaches (Matrane et al., 2023).

In a lexicon-based technique, sentiment analysis operates by giving a polarity score to each token in the text. The ratings are then averaged, with positive, negative, and neutral values tallied individually. The overall polarity of the text is ascertained by identifying the greatest value among the various scores. (Elshakankery and Ahmed 2019) introduced HILATSA, a hybrid incremental learning method that combines a lexicon-based approach with machine learning. The system updates its sentiment lexicon incrementally with newly labeled data. On the ArTwitter and Syria_Tweets datasets, it achieved an accuracy of 85% (SVM) and 75.5% (RNN), respectively.

(Abdulla et al. 2013) conducted an initial study on Arabic sentiment analysis, comparing lexicon-based and corpus-based methodologies. In the lexicon-based technique, an Arabic sentiment lexicon was manually created by expanding a set of seed words and assigning polarity ratings, thereafter categorizing text based on the aggregate sentiment of its words. Their study used a manually annotated dataset of 2,000 Arabic social media comments and reviews, which underwent preprocessing using light stemming approaches. The lexicon-based technique achieved an accuracy of around 59%, demonstrating the feasibility of rule-based sentiment classification in the absence of huge labeled datasets, while also highlighting its dependence on the comprehensiveness and quality of the lexicon.

(Mataoui et al. 2016) focused on vernacular Algerian Arabic, creating three dialect-specific sentiment lexicons and a manually annotated dataset sourced from social media. Their lexicon-based algorithm sorted texts by adding up the polarity of related phrases, which was around 61% accuracy. This shows that rule-driven methods may work well in very dialectal settings, but they also depend on having a complete vocabulary. (Assiri et al. 2018) enhanced lexicon-based sentiment analysis for the Saudi Arabic dialect by creating a comprehensive dialectal lexicon and using weighted polarity scoring that accounts for negation and supplication. Their method got around 68% of the answers right on a Saudi social media dataset, which is better than standard lexical baselines.

Machine learning approaches have also been applied to ASA. This approach is based on an annotated corpus, which is fed into ML algorithms in the training phase; then, after the model is trained, unannotated sentences are fed to the model to predict their polarity. (Aladeemy et al. 2024) applied a range of traditional machine learning algorithms—namely SVM, Random Forest, Decision Tree, Logistic Regression, and XGBoost—using BoW and TF-IDF representations with unigram and bigram features. The best result was achieved by SVM, with an accuracy of 90.3% using unigram features.

(Tubishat et al. 2019) proposed an Improved Whale Optimization Algorithm (IWO for feature selection in Arabic sentiment analysis. Their method integrates Elite Opposition-Based Learning to improve population diversity and Differential Evolution operators to refine the optimization process. The proposed approach was tested on four datasets and yielded a best average accuracy of 89.68% on the ArTwitter dataset. However, the introduction of pre-trained word embeddings brought a notable shift. For example, (Gamal et al. 2019) introduced a Twitter benchmark dataset for ASA and showed that distributed word representations capture semantic context far better than traditional bag-of-words features, even for short and noisy tweets.

A more recent trend has been targeted sentiment analysis (TSA), which focuses on detecting sentiment toward a specific entity within a text. In this area, (Sahmoud et al. 2022) released AT-ODTSA, a large-scale dataset of Arabic tweets annotated for open-domain TSA. This dataset spans multiple topics and sentiment targets, making it a valuable resource for fine-grained sentiment studies. However, our work differs in scope: we focus on overall tweet-level sentiment classification, applying and evaluating models on both a balanced dataset (ArTwitter) and a highly imbalanced one (Syria_Tweets).

Lately, transformer-based models have also entered the scene. For example, (Alsalem and Abudalfa 2024) fine-tuned AraBERT for Arabic sentiment tasks, achieving impressive results but requiring significant computational resources. Likewise, a recent study (Alosaimi et al. 2024) explored hybrid pipelines that combine pre-trained embeddings with traditional classifiers for low-resource languages. While promising, these works did not deeply investigate imbalanced Arabic datasets or compare classical ensemble methods under such conditions.

In contrast, our study combines multiple pre-trained embeddings with a homogeneous hard-voting ensemble of classical classifiers, and evaluates performance on both balanced and imbalanced datasets. We also address imbalance directly using SMOTE and report results using both accuracy and F1-score, allowing for a fairer and more informative comparison with recent state-of-the-art methods.

Ensemble Machine learning was applied by (Saleh et al. 2022), which developed a heterogeneous stacking ensemble model that combines RNN, LSTM, and GRU as base learners with meta-learners such as Logistic Regression, Random Forest, and SVM. Using CBOW features, their model attained an accuracy of 83.12% on the ArTwitter dataset. (Al-Azani and El-Alfy 2017) employed word2vec embeddings combined with single and ensemble machine learning classifiers to handle highly imbalanced sentiment datasets. They applied SMOTE for data balancing and reported their best result—80% accuracy—using the KNN classifier on the Syria_Tweets dataset.

While previous research has explored a range of lexicon-based, machine learning, deep learning, and ensemble techniques for Arabic sentiment analysis, most studies have either focused on a single dataset, relied heavily on deep neural models with high computational demands, or overlooked the performance implications of dataset imbalance. Our work distinguishes itself by systematically evaluating a homogeneous hard-voting ensemble of classical classifiers in combination with multiple pre-trained Arabic word embeddings. This design leverages the semantic richness of modern embeddings while retaining the efficiency and interpretability of traditional algorithms. Furthermore, by conducting experiments on both a balanced dataset (ArTwitter) and a highly imbalanced dataset (Syria_Tweets), and applying SMOTE to mitigate imbalance, we provide a more comprehensive assessment of model robustness.

## 3 Materials and methods

**Figure 1:**
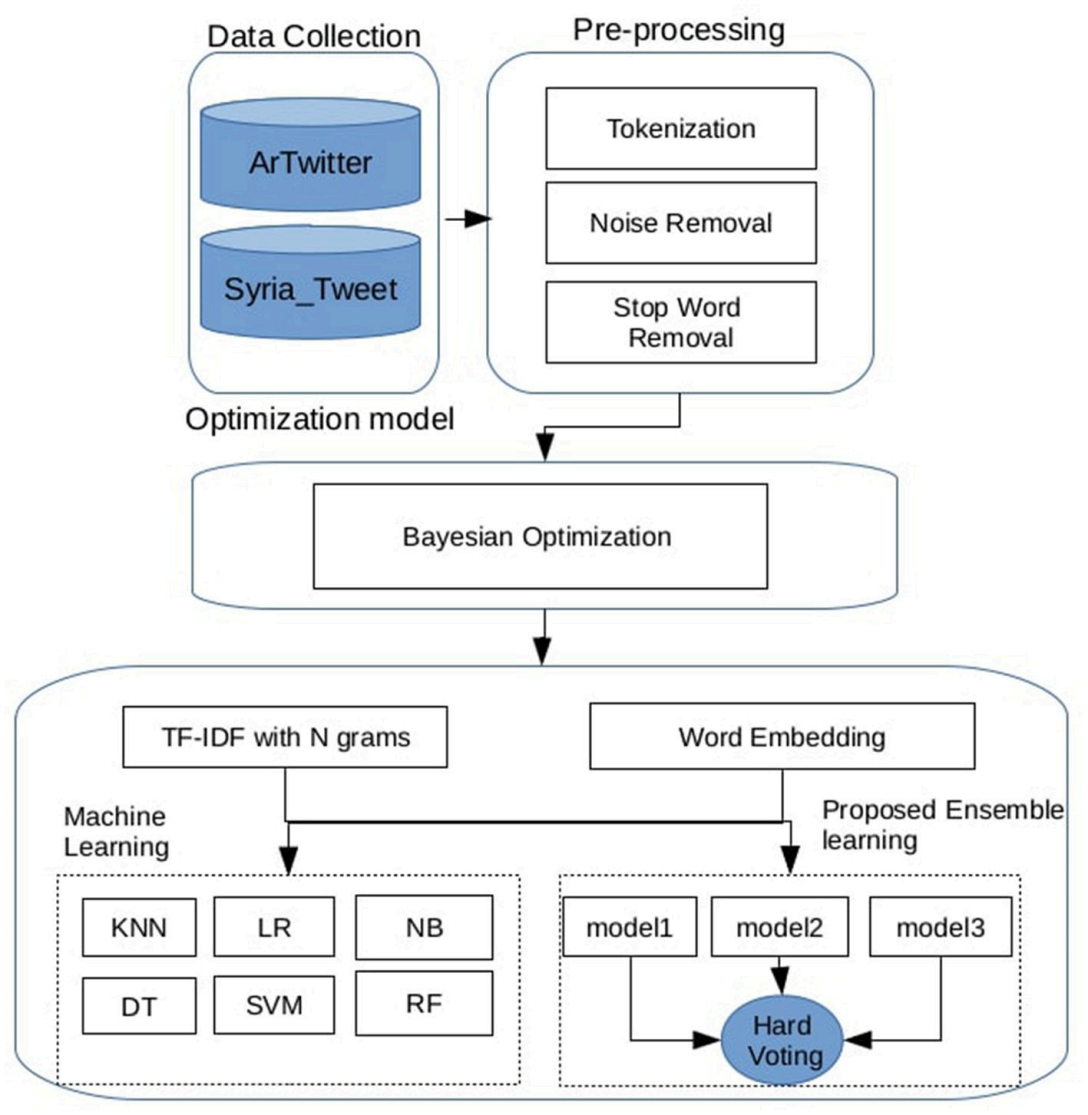
Architecture of the proposed arabic sentiment analysis framework.

An overview of the proposed Arabic Sentiment Analysis Framework is illustrated in Figure 1. The process begins with dataset preprocessing, which includes several text-cleaning steps. The textual data is then transformed into numerical vectors using two feature engineering techniques: the first involves TF-IDF with n-gram representations, and the second leverages the averaged vectors of pre-trained Word2Vec embeddings. A set of individual machine learning classifiers is subsequently trained, with their hyperparameters optimized using Bayesian optimization. Finally, several hard voting ensemble models are constructed by combining different classifiers to improve overall performance. The following subsections provide a detailed explanation of each step in the proposed pipeline.

### 3.1 Dataset

This study employed two sets of data. The ArTwitter dataset, created by (Abdulla et al. 2013), is a balanced corpus focusing on Modern Standard Arabic (MSA). Two thousand tweets of various topics, such as politics and arts, were gathered from Twitter and completely labeled by specialists in the field as either positive or negative. ArTwitter has been commonly used as a standard dataset in Arabic sentiment analysis research since it is balanced and includes high-quality annotations. The second data set is a highly unbalanced data set, which the Twitter API acquired from Syrian tweets in May 2014. Syria_Tweets (Mohammad et al., 2016) composed from 1,798 tweets; 1,350 are annotated as negative tweets and 448 are annotated as positive tweets. Table 1 illustrates the key characteristics of the used data sets.

**Table 1:** Key characteristics of the ArTwitter and Syria_Tweets sentiment analysis datasets.

**Feature**	**ArTwitter**	**Syria_Tweets**
Source	Twitter	Twitter
Language variety	Modern Standard Arabic (MSA)	Levantine dialectal Arabic
Annotation	Manually annotated	Manually annotated
Total tweets	1,951	1,798
Sentiment classes	Positive, negative	Positive, negative
Positive samples	993	1,350
Negative samples	958	448

### 3.2 Data set preprocessing

An essential phase is the preprocessing of the dataset, which guarantees that the data is clean, standardized, and fit for sentiment analysis. Due to the complexities of the Arabic language, this process employs various tailored methods to improve the dataset's quality and ensure that the text is well-prepared for both machine learning and ensemble learning models. The preprocessing pipeline initially involves the removal of NaN values and duplicates to uphold data integrity. Following this, the text undergoes systematic cleaning to tackle important linguistic challenges such as punctuation and inconsistencies in spelling and writing styles. Standardization techniques, such as removing punctuation and normalizing text, aid in unifying the data, thereby enhancing model accuracy. Further cleaning procedures are implemented to remove noise and irrelevant elements, such as non-Arabic characters, emojis, and English words or numbers. These actions ensure that only pertinent information is retained, thus optimizing the dataset for sentiment classification. The preliminary data cleaning operations, which were performed by using the NLTK library (Bird et al., 2009) and the ISRI Arabic stemmer (Taghva et al., 2005), include:

- **Stopword removal:** removing common words like conjunctions (e.g: ثم) and (e.g: في, من, الى), which have little semantic importance and do not meaningfully assist in classification efforts.
- **Punctuation removal:** stripping punctuation from Arabic text to reduce extraneous data and simplify further analysis (e.g:?, !, ...).
- **Hashtag and mention removal:** eliminating hashtags and user tags (like @username, #hashtag) from the text.
- **Emoji removal:** extracting emoji characters using a regular expression pattern to cleanse the dataset by matching and eliminating emojis.
- **English words and numbers removal:** taking out English terms and numerals from the Arabic script using regular expressions to identify and discard typical alphanumeric sequences.
- **Character repetition handling:** reducing sequences of repeated characters to a single character (e.g: ههههههههه, اللهههه).
- **Whitespace cleanup:** compressing multiple spaces into a single space for text uniformity.
- **Tokenization:** this step breaks down the polished text into discrete tokens or units by employing separator characters such as spaces, commas, or tabs, facilitating separate analysis of each word or element.

### 3.3 Data balancing technique

An imbalanced dataset is characterized by an unequal distribution of class labels, where the majority class comprises a large number of training samples, and the minority class contains relatively few annotated instances. To address this issue, the *Synthetic Minority Oversampling Technique (SMOTE)* (Chawla et al., 2002) is one of the most widely adopted solutions.

SMOTE improves the representation of the minority class by generating synthetic samples based on the feature space similarities between existing instances. For each minority class instance *x*_*i*_ ∈ *S*_min_, SMOTE identifies its *k*-nearest neighbors (typically using Euclidean distance), and constructs synthetic examples by linearly interpolating between *x*_*i*_ and one of its neighbors. Specifically, a new sample is generated as:


(1) $$\begin{array}{l} x_{\text{new}}=x_{i}+\delta \cdot \left(x_{nn}-x_{i}\right) \end{array}$$

where *x*_*nn*_ is one of the *k*-nearest neighbors of *x*_*i*_, and δ ∈ [0, 1] is a random number. This interpolation ensures that the synthetic instances are consistent with the local topology of the minority class (He and Garcia, 2009). The oversampling process continues until the minority class is balanced or reaches a predefined target size. In our study, we applied SMOTE with *k* = 5 nearest neighbors. SMOTE technique was applied only to the training set, while the testing sets remained unbalanced, to maintain the original class distribution.

### 3.4 Feature representation methods

Transforming text into numerical values while representing the semantic meaning of the text is the nex step after the cleaning of the data. In this work, several forms of N-grams with TF-IDF representations were implemented, in addition to pre-trained word embedding with word2vec was leveraged to improve the performance of the proposed models. In the following subsections a brief descriptions for the data representation methods that were used in the study.

#### 3.4.1 TF-IDF with n-grams

Term Frequency-Inverse Document Frequency (TF-IDF) is a common way to weight words and phrases in text classification. It looks at how important a word or phrase is in a document compared to a group of documents. It balances out two things: word Frequency (TF), which counts how many times a word appears in a text, and Inverse Document Frequency (IDF), which makes common words less important and puts greater emphasis on unique phrases. The TF-IDF score is calculated as:


(2) $$\begin{array}{l} \text{TF-IDF}\left(t,d\right)=\text{TF}\left(t,d\right)\times \log\left(\frac{N}{\text{DF}\left(t\right)}\right) \end{array}$$

where *t* is the term, *d* is the document, *N* is the total number of documents, and DF(*t*) is the number of documents containing term *t*. To capture local context and word co-occurrence patterns, we applied TF-IDF weighting over n-gram features.

N-grams (Jurafsky and Martin, 2009) represent one of the simplest and most widely used approaches to language modeling in natural language processing. They are used to represent textual data by capturing contiguous sequences of words. A single word forms a unigram, a sequence of two consecutive words is referred to as a bigram, and a sequence of three successive words is known as a trigram. Despite their simplicity, n-gram models effectively capture local context and are commonly used in various tasks such as text classification, sentiment analysis, and machine translation. Table 2 shows an example of how the sentence is tokenized based on the chosen type of n-grams.

**Table 2:** N-gram generation examples for feature extraction.

**N-gram**	**Results**
*Original Arabic Sentence*	[عمر انت مميز جد رائع]
*Unigram*	[عمر], [انت], [مميز], [جد], [رائع]
*Bigram*	[عمر انت], [انت مميز], [مميز جد], [جد رائع]
*Trigram*	[عمر انت مميز] , [انت مميز جد] , [مميز جد رائع]

In our study, we examined the effectiveness of three types of n-gram features—unigram, bigram, and trigram—in combination with machine learning and ensemble learning approaches.

#### 3.4.2 Pre-trained word embeddings

ArWordVec (Fouad et al., 2020) is a huge set of pretrained models that is built from 55 million tweets with different topics, including social affairs, politics, and health care. The embeddings are trained by word2vec and Glove methods with different approaches, window size, and vector size.

In our experiments, we used the Word2Vec architecture with the Skip-Gram (SG) approach, a window size of 3, and an embedding dimension of 300. The Skip-Gram model was chosen because it tends to perform better with infrequent words and is more effective at capturing detailed semantic relationships than the Continuous Bag-of-Words (CBOW) method (Mikolov et al., 2013a). A relatively small window size of 3 was selected to emphasize local contextual dependencies, which suits the characteristics of the used dataset, while limiting the influence of less relevant, distant words. The choice of a 300-dimensional vector is consistent with common practice in earlier studies (Mikolov et al., 2013b; Pennington et al., 2014), as it offers a practical balance between the ability to represent nuanced meaning and the need to keep training time and memory use manageable.

To leverage the strengths of the model, we compute the average of the word embedding vectors across the entire sentence, as defined in Equation 3.


(3) $$\begin{array}{l} \text{AVG}\left(E\left(S\right)\right)=\frac{1}{n}\overset{n}{\underset{i=1}{\sum }}\text{Emb}\left(S\left(i\right)\right) \end{array}$$

Where AVG(E(S)) is the average embedding of the sentence S, S(i) is the i-th word in the sentence, Emb(S(i)) is the embedding of word i, and n is the total number of words in the sentence.

### 3.5 Individual machine learning models

Several individual Machine learning classifiers were implemented. A brief definition of the selected algorithms is provided below:

- Naïve Bayes (NB) (Duda et al., 2001): is a probabilistic classifier that uses Bayes' theorem and assumes that features are very independent of each other. Even though it's simple, it does an amazing job at classifying text because it's fast and works well with data that has a lot of dimensions.
- Support Vector Machine (SVM) (Cortes, 1995): builds the best hyperplane to divide classes with the most space between them. This makes it work well in spaces with a lot of dimensions. It is considered powerful due to its kernel functions that work well for non-linear decision boundaries.
- Stochastic Gradient Descent (SGD) (Bottou, 2010): it is a good choice for sparse datasets, it updates its model parameters in an iterative optimization process for linear classifiers.
- Logistic Regression (LR) (Cox, 1958): logistic functions are used to model of the probability of binary results.
- Random Forest (RF) (Breiman, 2001): builds multiple decision trees and combines their results to enhance generalization and decrease overfitting.

### 3.6 Ensemble learning models

Ensemble learning aims to optimize the classification task by fusing multiple base classifiers, which reduces the variance of the predictions of the individual classifiers (Kumar et al., 2020). Thus, several ensemble techniques are designed to achieve this goal, such as bagging (Yang et al., 2020), boosting (Deng et al., 2023), and voting (Onan et al., 2016).

The use of heterogeneous base classifiers is utilized in the Voting technique for the production of concurrent ensemble networks. Voting is categorized into two types: weighted averaging and majority voting, which this study uses.

In majority voting, each model “votes” for a class label; the most voted label is chosen for the final predictions. This happens by combining several individual classifiers, which are known as base learners, and the majority vote makes the final decision. In this study, combinations of sets of individual machine learning classifiers were tested, it is named v with numbers from 1 to 11.

### 3.7 Evaluation metrics

To measure the performance of the proposed approaches, two datasets were used with different setups. We performed an 80/20 train-test split using stratified sampling, ensuring that both subsets maintained the original class imbalance of approximately 75% negative and 25% positive tweets. SMOTE was applied only to the training set, while the test set remained untouched to evaluate model performance on real-world imbalanced data. The vectorized training and test datasets were input into the Machine learning classifiers in addition to ensemble learning.

The machine learning classifiers were trained to determine the sentiment polarity of the reviews as either positive or negative. To evaluate model performance, we used four standard classification metrics: precision, recall, F-measure, and accuracy. These are defined in Equations 4–7.


(4) $$\begin{array}{l} \text{Precision}=\frac{TP}{TP+FP} \end{array}$$

(5) $$\begin{array}{l} \text{Recall}=\frac{TP}{TP+FN} \end{array}$$

(6) $$\begin{array}{l} F\text{-measure}=2\times \frac{\text{Precision}\times \text{Recall}}{\text{Precision}+\text{Recall}} \end{array}$$

(7) $$\begin{array}{l} \text{Accuracy}=\frac{TP+TN}{TP+TN+FP+FN} \end{array}$$

where *TP*, *TN*, *FP*, and *FN* represent true positives, true negatives, false positives, and false negatives, respectively.

## 4 Experiments results and discussion

### 4.1 Experiments setup

All experiments were performed on the Google Colab platform, utilizing a Tesla T4 GPU for accelerated computation mainly for faster processing of the embedding and hyperparameter tuning. After data set preprocessing, the data was split into 80% training and 20% testing data sets. Then, the SMOTE technique was applied to the Syria_tweet dataset to solve the imbalanced dataset problem. SMOTE techniques were applied to the training dataset to make sure the learned model would be tested on real test data.

#### 4.1.1 Hyperparameter optimization

For optimizing the performance of the proposed models, Bayesian Hyperparameter optimization techniques (Snoek et al., 2012) were applied to both TF-IDF with n-grams and word embeddings feature extractions.The optimization techniques were applied via the Gaussian Process-based. This method models the objective function using a Gaussian Process, which provides uncertainty estimates that guide the search efficiently through the hyperparameter space. We set the number of iterations to 32 and employed three-fold cross-validation. As shown in Table 3, the optimal hyperparameter values vary between the two datasets. For example, the alpha parameter in Naive Bayes is smaller for the Syria_Tweets dataset compared to ArTwitter. Additionally, the SVM model uses a linear kernel for ArTwitter, while an RBF kernel is preferred for Syria_Tweets.

**Table 3:** Best hyperparameters for ArTwitter and Syria_Tweets datasets across TF-IDF with N-gram models.

**Classifier**	**Hyperparameter**	**Unigram**		**Bigram**		**Trigram**	
		**ArTwitter**	**Syria**	**ArTwitter**	**Syria**	**ArTwitter**	**Syria**
Naive Bayes (NB)	Alpha	0.0340	0.0010	0.0275	0.0010	0.1896	0.0010
SVM	C	0.9635	3.6975	0.4667	105.7621	0.6839	105.7621
	Gamma	0.0015	0.0271	0.0570	0.0447	0.1	0.0447
	Kernel	Linear	Linear	Linear	Rbf	Linear	Rbf
KNN	Metric	Minkowski	manhattan	Minkowski	Manhattan	Euclidean	Manhattan
	n_neighbors	12	2	14	2	4	2
	Weights	Uniform	Uniform	Uniform	Uniform	Uniform	Uniform
Decision Tree (DT)	MAX_depth	39	35	50	21	50	32
	Min_samples_leaf	1	1	1	1	1	1
	Min_samples_split	20	2	19	2	15	3

Table 4 shows the optimal values of the hyperparameters for different sets of machine learning algorithms after applying Bayesian optimization.

**Table 4:** Best hyperparameters using Word2Vec for ArTwitter and Syria_Tweets datasets.

**Classifier**	**Hyper- parameter**	**ArTwitter value**	**Syria_Tweets value**
SGD	Alpha	1e-06	0.000563
	eta0	1.0225	0.0174
	Learning_rate	Invscaling	Adaptive
	Loss	Log_loss	Log_loss
	Max_iter	3251	1000
	Penalty	Elasticnet	l1
	Tol	0.01	1.41e-05
Logistic regression (LR)	C	0.5023	11185.625
	Penalty	l2	l2
	Solver	Liblinear	Liblinear
Support vector machine (SVM)	C	25.8455	30.0
	Gamma	0.1877	0.15
	Kernel	rbf	rbf
K-Nearest Neighbors (KNN)	Metric	Minkowski	Manhattan
	n_neighbors	6	2
	Weights	Uniform	Uniform
Random Forest (RF)	Bootstrap	False	False
	Max_depth	50	45
	Max_features	Log2	Sqrt
	Min_samples_leaf	1	1
	Min_samples_split	2	2
	n_estimators	500	500

It's important to note that the tuning parameters are very different between the two datasets. For example, SGD hyperparameters optimized for ArTiwtter data set in a much smaller learning rate initialization (eta0) and used a “invscaling” learning schedule with a elasticnet penalty. While Syria_Tweets hyperparameters optimized to an “adaptive” schedule and an “l1” penalty,An adaptive learning rate helped keep the model's training on a stable and efficient path. At the same time, the L1 penalty was great at promoting feature sparsity, which let the model focus on the most important predictors and tune out the noise in the data, preventing it from just memorizing the training examples. . However, the SVM classifier shared the same RBF kernel across both datasets. The KNN classifier revealed greater variation: ArTwitter favored six neighbors and the Minkowski distance, while Syria_Tweets performed best with just two neighbors and the Manhattan distance, indicating that Syria_Tweets required tighter local decision boundaries.

### 4.2 Results

Table 5 presents the performance of both individual and ensemble learning models using TF-IDF with unigram, bigram, and trigram representations on the ArTwitter dataset. The results demonstrate that unigram features consistently outperform both bigram and trigram configurations. Among the individual classifiers, Naive Bayes (NB) achieved the highest accuracy of 89.27 and 89.00% F1-score with unigrams, followed closely by SVM with 88.01% accuracy and 88.0% F1-score. Notably, all ensemble models outperformed the individual classifiers across the different n-gram representations. The V1 ensemble model (comprising NB, SVM, and DT) achieved the highest accuracy of 90.22 and 90.00% F1-score with unigram features, highlighting the effectiveness of combining diverse classifiers.

**Table 5:** Performance across unigram, bigram, and trigram features on the ArTwitter dataset.

**Classifier**	**Unigram**				**Bigram**				**Trigram**			
	**Acc()**	**Prec**.	**Rec**.	**F1**	**Acc**.	**Prec**.	**Rec**.	**F1**	**Acc**.	**Prec**.	**Rec**.	**F1**
NB	89.27	89.00	89.00	89.00	87.70	88.00	88.00	88.00	86.75	87.00	87.00	87.00
SVM	88.01	88.00	88.00	88.00	86.75	87.00	87.00	87.00	84.54	85.00	85.00	85.00
K-NN	83.91	84.00	84.00	84.00	81.39	82.00	81.00	81.00	80.44	80.00	80.00	80.00
DT	79.18	80.00	79.00	79.00	81.70	82.00	82.00	82.00	81.70	82.00	82.00	82.00
V1 (NB, SVM, DT)	**90.22**	90.00	90.00	90.00	**89.27**	89.00	89.00	89.00	**88.96**	89.00	89.00	89.00
V2 (NB, SVM, K-NN)	89.91	90.00	90.00	90.00	87.38	87.00	87.00	87.00	83.60	84.00	84.00	83.00
V3 (NB, DT, K-NN)	88.01	88.00	88.00	88.00	87.70	88.00	88.00	88.00	86.75	87.00	87.00	87.00
V4 (SVM, DT, K-NN)	88.01	88.00	88.00	88.00	87.38	88.00	87.00	87.00	85.17	0.85	85.00	85.00

Bold values indicate the best performance of each model.

For the balanced Syria_Tweets dataset, Table 6 reveals more consistent performance across all n-gram representations. Both NB and SVM classifiers showed strong results, achieving 81.47 and 81.76% accuracy, respectively, using unigram features and 80.69% and 81.25 F1-score. However, ensemble models again demonstrated superior performance. In particular, the V4 ensemble (SVM, DT, and KNN) achieved the highest accuracy of 83.82 and 83.33% F1-score with bigram features, indicating that ensemble learning can capture richer contextual information and provide more robust classification in complex datasets.

**Table 6:** Performance across unigram, bigram, and trigram features on the Syria_Tweets dataset.

**Classifier**	**Unigram**				**Bigram**				**Trigram**			
	**Acc**.	**Prec**.	**Rec**.	**F1**	**Acc**.	**Prec**.	**Rec**.	**F1**	**Acc**.	**Prec**.	**Rec**.	**F1**
NB	81.47	80.43	81.47	80.69	81.18	80.06	81.18	80.33	81.76	80.79	81.76	81.05
SVM	81.76	80.99	81.76	81.25	80.88	79.88	80.88	80.19	81.18	80.15	81.18	80.44
K-NN	80.29	79.36	80.29	79.69	79.71	79.00	79.71	79.29	78.82	80.18	78.82	79.36
DT	79.41	77.99	79.41	78.37	79.71	77.94	79.71	78.07	80.00	79.38	80.00	79.64
V1 (NB, SVM, DT)	**83.53**	82.64	83.53	82.14	82.65	81.54	82.65	81.24	**83.24**	82.26	83.24	81.88
V2 (NB, SVM, K-NN)	82.65	81.66	82.65	81.82	82.06	81.08	82.06	81.31	81.18	80.15	81.18	80.44
V3 (NB, DT, K-NN)	82.35	81.44	82.35	81.66	82.94	82.44	82.94	82.63	80.88	81.13	80.88	81.00
V4 (SVM, DT, K-NN)	82.65	81.66	82.65	81.82	**83.82**	83.14	83.82	83.33	82.35	82.21	82.35	82.28

Bold values indicate the best performance of each model.

Finally, Table 7 presents the results of individual and ensemble models using word embeddings on both datasets. Across the board, word embeddings improved the performance of all models compared to the TF-IDF-based representations. Ensemble models significantly outperformed individual classifiers in both datasets. On the ArTwitter dataset, the V4 ensemble (SGD, SVM, RF) achieved the highest accuracy of 92.43% 92.00% F1-score. On the Syria_Tweets dataset, the best performance was obtained by the V5 ensemble (SGD, KNN, RF), which reached an accuracy of 83.82% 83.86% F1-score. These findings confirm the effectiveness of combining rich semantic features with ensemble strategies to enhance classification accuracy in Arabic social media text.

**Table 7:** Individual classifiers and ensemble performance using word embeddings on ArTwitter and balanced Syria_Tweets datasets.

**Classifier**	**ArTwitter**				**Syria_Tweets**			
	**Accuracy (%)**	**Precision**	**Recall**	**F1-score**	**Accuracy (%)**	**Precision (%)**	**Recall (%)**	**F1-score (%)**
SGD	89.27	89.00	89.00	89.00	79.12	81.69	79.12	79.97
LR	**90.54**	91.00	91.00	91.00	75.59	78.89	75.59	76.70
SVM	**90.54**	91.00	91.00	91.00	80.85	81.00	80.85	80.90
K-NN	84.20	86.00	83.00	84.50	76.18	81.33	76.18	77.57
RF	88.96	89.00	89.00	89.00	81.76	80.49	81.76	80.48
V1 (SGD, LR, SVM)	92.11	92.00	92.00	92.00	82.50	83.00	82.50	82.60
V2 (SGD, LR, K-NN)	91.10	91.80	91.10	91.30	79.41	81.85	79.41	80.22
V3 (SGD, LR, RF)	91.17	91.00	91.00	91.00	79.12	80.57	79.12	79.68
V4 (SGD, SVM, RF)	**92.43**	92.00	92.00	**92.00**	82.10	82.40	82.10	82.20
V5 (SGD, K-NN, RF)	91.85	91.70	91.60	91.65	**83.82**	83.89	83.82	**83.86**
V6 (LR, SVM, RF)	91.48	92.00	91.00	91.00	82.60	82.90	82.60	82.70
V7 (LR, K-NN, RF)	91.00	91.30	91.00	91.10	83.24	83.17	83.24	83.20

Bold values indicate the best performance of each model.

### 4.3 Error analysis

To gain a clearer picture of where our model falls short, we looked closely at tweets it misclassified in both datasets. Three main patterns stood out.

First, sarcasm and irony often tripped the model. Tweet التعدد جميل جداً ولكن يحتاج إلى كثييييييييييير من المال which means in English “Polygamy is very beautiful, but it requires a lot of money.” used positive wording to express criticism, usually labeled incorrectly because the model lacked any mechanism to detect sarcasm. Second, dialectal variation posed a challenge. Like tweet “ لا تتحمس وايد بموضوع (العدل) لأن الشي مب سهل” which means in English “Don't get too excited about the topic of it's not easy.” The tweet contained regional expressions, particularly from Gulf “مب,وايد,” that were not well captured in the embeddings. Words that carried a negative tone in one dialect could be interpreted as neutral in another, leading to incorrect predictions.

Finally, mixed sentiment such as انا مع حقوق المرأة دايما لكن مستحيل أقول عن نفسي نسوية which means in English “I am always for women's rights, but it is impossible for me to call myself a feminist.” The tweet conveyed both positive and negative feelings about different entities were often reduced to a single overall sentiment, which meant losing important nuances. A more fine-grained, aspect-based approach would likely handle such cases better.

### 4.4 Comparison of the proposed model with existing work

To compare the proposed approach with the most relevant previous studies, Table 8 presents the results of selected works. (Al-Saqqa et al. 2018) applied ensemble learning using traditional machine learning classifiers and achieved an accuracy of 84.4%. (Saleh et al. 2022) employed a stacking ensemble method that integrated deep learning architectures such as RNN, LSTM, and GRU, with an SVM meta-classifier, achieving 83.12% accuracy. The most recent work by (Aladeemy et al. 2024) attained 90.3% accuracy using a standalone SVM classifier with unigram features. In contrast, our proposed approach—based on hard voting ensemble learning that combines SGD, SVM, and Random Forest classifiers with pre-trained word embeddings—achieved the highest accuracy of 92.43%, demonstrating its superior performance in Arabic sentiment classification.

**Table 8:** Comparison of accuracy between previous and our study on ArTwitter Dataset.

**Reference**	**Approach**	**Accuracy**	**F1 score**
(Al-Saqqa et al. 2018)	Ensemble machine learning (voting of KNN, SVM, DT, NB)	84.4% (SVM individually)	84.0%
(Saleh et al. 2022)	Stacked deep learning (RNN, LSTM, GRU + SVM meta-learner)	83.12%	82.8%
(Aladeemy et al. 2024)	Machine learning (SVM with BoW Unigram)	90.3%	90.3%
Our approach	Ensemble machine learning (voting of SGD, SVM, RF)	**92.43%**	92.0%

Bold values indicate the best performance of each model.

However, related to the Syria_Tweet data set, the F1-score is used because the accuracy isn't available. Table 9 compares our results with the most related previous work. As shown, our approach with ensemble voting (SGD, K-NN, RF) improved the performance of analyzing the sentiment of the dataset. The ensemble stacking approach was applied on the same data set by (Al-Azani and El-Alfy 2017), and the F1-score achieved is 63.95%. While a traditional ML algorithm, which is SGD, was applied by (El-Alfy and Al-Azani 2020) and achieved a 70.7% F1-score.

**Table 9:** Comparison of F1 score between previous and our study on Syria_Tweets Dataset.

**Reference**	**Approach**	**F1-score**
(Al-Azani and El-Alfy 2017)	Ensemble machine learning (stacking)	63.95%
(El-Alfy and Al-Azani 2020)	Machine learning (SGD classifier)	70.7%
Our approach	Ensemble machine learning (voting of SGD, K-NN, RF)	**83.86%**

Bold values indicate the best performance of each model.

## 5 Conclusion and future direction

The objective of this study was to investigate multiple methodologies for feature extraction specifically tailored for Arabic sentiment analysis. Our focus was directed toward analyzing three distinct types of n-gram features—namely, unigram, bigram, and trigram—alongside leveraging a pre-trained Word2Vec word embedding model. A diverse machine learning algorithms was employed in our analysis, including Support Vector Machines (SVM), k-Nearest Neighbors (K-NN), Stochastic Gradient Descent (SGD), Logistic Regression (LR), and Random Forest (RF). Additionally, we implemented ensemble techniques based on hard voting.

The experimental investigations were conducted utilizing two distinct datasets: the balanced ArTwitter dataset and the significantly imbalanced Syria_Tweets dataset. To address the issue of class imbalance present in the Syria_Tweets dataset, the Synthetic Minority Oversampling Technique (SMOTE) was applied during the training phase.

Our results indicated that Naïve Bayes (NB) achieved the highest accuracy rate of 89.79 and 89% F1-score on the ArTwitter dataset when unigram features were employed. Conversely, the Support Vector Machine (SVM) achieved an accuracy rate of 81.76 and 81.25% F1-score on the Syria_Tweets dataset, with SVM excelling with unigram features and NB performing optimally with trigram features. Notably, the hard voting ensemble containing Naïve Bayes (NB), Support Vector Machine (SVM), and Decision Tree (DT) utilizing unigram features outperformed others on the ArTwitter dataset, achieving an accuracy of 90.22% and 90% F1-score. Meanwhile, the hard voting ensemble combining SVM, DT, and K-Nearest Neighbors (K-NN) attained superior results on the Syria_Tweets dataset with an accuracy of 83.82% and 83.33% F1-score when employing bigram features. However, average weighted pretrained word embedding achieved superior results on both datasets with the ensemble approach; hard voting (SGD, SVM, and RF) achieved 92.43% accuracy and 92% F1-score on ArTwitter Dataset. While hard voting (SGD, KNN, and RF) achieved 83.82% accuracy and 83.86% F1-score on Syris_tweet dataset.

The outcomes of this research suggest that leverage pretrained word embedding in representing the data can significantly enhance model performance and that ensemble approaches contribute to a more robust overall system. Looking ahead, there is potential for employing transformer-based models, which provide deep contextualized embeddings, thereby further optimizing performance. The exploration of novel data balancing methodologies could advance the efficacy of model operation.

## Data Availability

The original contributions presented in the study are included in the article/supplementary material, further inquiries can be directed to the corresponding author.
